# Factors associated with an unsuppressed viral load among HIV-positive individuals attending STI services in South Africa, 2019

**DOI:** 10.1186/s12879-023-08756-1

**Published:** 2024-01-30

**Authors:** Marceline Mapiye, Khuliso Ravhuhali, Alex de Voux, Tendesayi Kufa

**Affiliations:** 1 Faculty of Health Sciences, School of Public Health, University of the Witwatersrand, Johannesburg, South Africa; 2 South African Field Epidemiology Training ProgrammeProgramme, National Institute for Communicable Diseases, National Health Laboratory Service, Johannesburg, South Africa; 3 Division of Epidemiology and Biostatistics, School of Public Health, University of Cape Town, Cape Town, South Africa; 4 Centre for HIV/STI, National Institute for Communicable Disease (NICD), Modderfontein, Johannesburg, South Africa

**Keywords:** HIV, Sexually Transmitted Infections, VL, VL suppression, South Africa, Antiretroviral therapy, 95–95-95

## Abstract

**Background:**

Sexually transmitted infections (STIs), particularly in the absence of viral suppression, increase the risk of HIV transmission to uninfected partners. We determined factors associated with having an unsuppressed VL among HIV-positive individuals attending STI services in South Africa (SA).

**Methods:**

We analysed secondary cross-sectional data collected on HIV-positive individuals presenting with STI symptoms s at sentinel sites in Western Cape and Gauteng provinces between January–December 2019 in SA. We compared demographic characteristics of individuals on ART or not on ART, and a Poisson regression model to identify factors associated with having an unsuppressed VL (≥ 50 copies/ml) was used.

**Results:**

Among 93 HIV-positive individuals attending STI services with VL data, the median age was 32 years (IQR 27–37). Thirty-two (34.41%) individuals were on ART compared to 61 (65.59%) not on ART. Most of those on ART (56.25%) had an unsuppressed VL, while 86.89% of those not on ART had an unsuppressed VL. ART use was associated with a 33% lower prevalence of having unsuppressed VL. In a model adjusting for age, age at first sex and oral sex, none of the factors were significant. Among those on ART, individuals < 25 years were more likely to have an unsuppressed VL (aPRR = 1.94: 95% CI = 1.27–2.97) compared to those ≥ 25 years.

**Conclusion:**

ART use among HIV-positive individuals was low and VL suppression among those on ART was sub-optimal. Intensified ART initiation and adherence support to HIV-positive individuals seeking STI services could improve VL suppression.

## Background

Sexually transmitted infection (STI) incidence has been increasing globally, mainly among HIV-positive adults [1]. In 2020, the World Health Organization’s (WHO) Global progress report on HIV and STIs estimated about 7.1 million people were newly infected with *Treponema pallidum* and 82.4 million people were newly infected with *Neisseria gonorrhoea* [1]. Sub-Saharan Africa bears about 40% of the global STI morbidity and mortality burden [2]. Huge progress has been made in improving access to HIV antiretroviral therapy (ART) through the universal test and treat (UTT) programme [3]. ART suppresses viral replication to reduce HIV transmission, however, despite ART access, viral outcomes may be compromised by infections such as STIs. STIs increase the risk of HIV transmission [4, 5]. South Africa (SA) adopted the 95-95-95 targets in which: By 2030, 95% of all HIV-positive patients should know their status by testing, 95% of HIV-positive patients should be on sustained ART and 95% of those on ART should have a suppressed HIV viral suppression. This study determined the prevalence of and factors affecting VL suppression among HIV-positive individuals attending STI services in South Africa in 2019.

## Methods

### Study design

We conducted a secondary analysis of cross-sectionaldata collected from the National Institute for Communicable Diseases (NICD) STI sentinel surveillance sites between January–December 2019 in South Africa.

### Data sources and study sites

The primary study aimed to evaluate and validate self-reported HIV testing, antiretroviral therapy (ART) use, and viral suppression measures among individuals attending STI services. The primary study enrolled consecutive individuals presenting with one or more symptoms of male urethritis (MUS), vaginal discharge syndrome (VDS), and genital ulcer syndrome (GUS) regardless of HIV status; aged ≥ 18 years and willing to consent to responding to study questions and genital and blood specimen collection. The study was conducted from 1 January to 31 December 2019 at two high-volume STI sentinel surveillance centres in Gauteng and Western Cape. STI sentinel surveillance sites were primary health centers selected to assess and monitor STI occurrences, causes and major risk groups among individuals. Relative to the 95–95–95 cascade measure in SA, Gauteng had an overall percentage of 86.5% of individuals knowing their HIV status and Western recorded 80.9%. Only 60.8% were on ART in Gauteng and 67.2% on ART in Western Cape. Both Gauteng and Western Cape were heading towards the 95% target with 87.7% and 89% with suppressed viral load (VL) respectively [3]. Consenting participants responded to a questionnaire collecting demographic, behavioural, and clinical data and provided genital specimens, endourethral or endocervical swabs and 10 ml whole blood specimens for laboratory testing. Specimens were tested according to HIV Sero-Molecular reference laboratory procedures. Enrolled participants had confirmatory HIV testing, VL testing, and pooled nucleic acid amplification testing (pNAAT) done. Data collected from the questionnaire and laboratory were linked through a barcode with a unique identifier which was sent to the STI Reference Centre at NHLS and double-captured into the Microsoft Access database.

### Study population

The study included all HIV-positive adults (aged ≥18 years) attending STI services (regardless of ART use) with VL clinical information and who had participated in the primary study in 2019. We excluded individuals who tested negative on the HIV confirmatory test and those without VL clinical information.

The research used all the data available (93 individuals) giving us limited power to detect associations between VL suppression among HIV-positive individuals attending STI services by ART status status.

### Data analysis

De-identified data were received for analysis in Microsoft Excel password-protected spreadsheets from the STI Reference Centre (NICD). Data were imported into STATA version 15 (STATA Corporation, College Station, TX, USA) for cleaning and analysis. The outcome variable was an unsuppressed VL analysed as a binary variable. A cut-off of ≥50 copies/ml was used as a threshold for unsuppressed VL. The explanatory variables used were gender (male or female), age in years as continuous, age groups (<25 and ≥25 years), age at first sex (<15 and ≥15 years), type/nature of sexual activity in the past 3 months categorized as none, vaginal (penis in vagina), oral (mouth on penis/ vagina/ anus) or receptive anal (penis in the rectum/anus) sex partner type in the last three months (casual, regular), number of sex partners in the last 3 months (one partner and >one partner), STI syndrome such as genital ulcer and discharge (VDS, MUS, GUS, none or missing), previous STI in the past 12 months (yes/no), condom use at last sexual encounter (yes/no), self-reported knowledge of HIV status (yes/no), time since recent HIV test (<3 months and ≥3 months) and self-reported ART use in the preceding 3 days (yes/no ).

Descriptive analysis was used to describe the demographic, sexual behaviour, and clinical characteristics to determine frequencies of categorical variables and medians and interquartile ranges (IQR) for continuous variables. The Chi-square and Fisher's exact test was used for cell counts with fewer than five observations to compare those on ART and those not on ART, a p-value less than 0.05 was considered statistically significant. The prevalence of having an unsuppressed VL was calculated as the proportion of individuals with a VL≥50 copies/ml among the total number of individuals with a VL result. For continuous variables, the Wilcoxon rank-sum test was used with a *p*-value < 0.05 considered significant.

To identify factors associated with an unsuppressed VL, a multivariable Poisson regression model with robust error variance was used [6]. Explanatory variables with a *p*-value of ≤0.25 from the univariable analysis were included in the multivariable model. In the multivariable model, variables with a *p*-value <0.05 were considered statistically significant [7]. A prevalence rate ratio greater than one (PRR>1) indicates a greater prevalence rate of having an unsuppressed VL while those with a PRR less than one (PRR<1) shows a smaller prevalence rate ratio of having an unsuppressed VL after adjusting for covariates. Furthermore, a sub-group multivariable analysis was done to determine factors associated with having an unsuppressed VL among only individuals on ART.

## Results

**Fig. 1:**
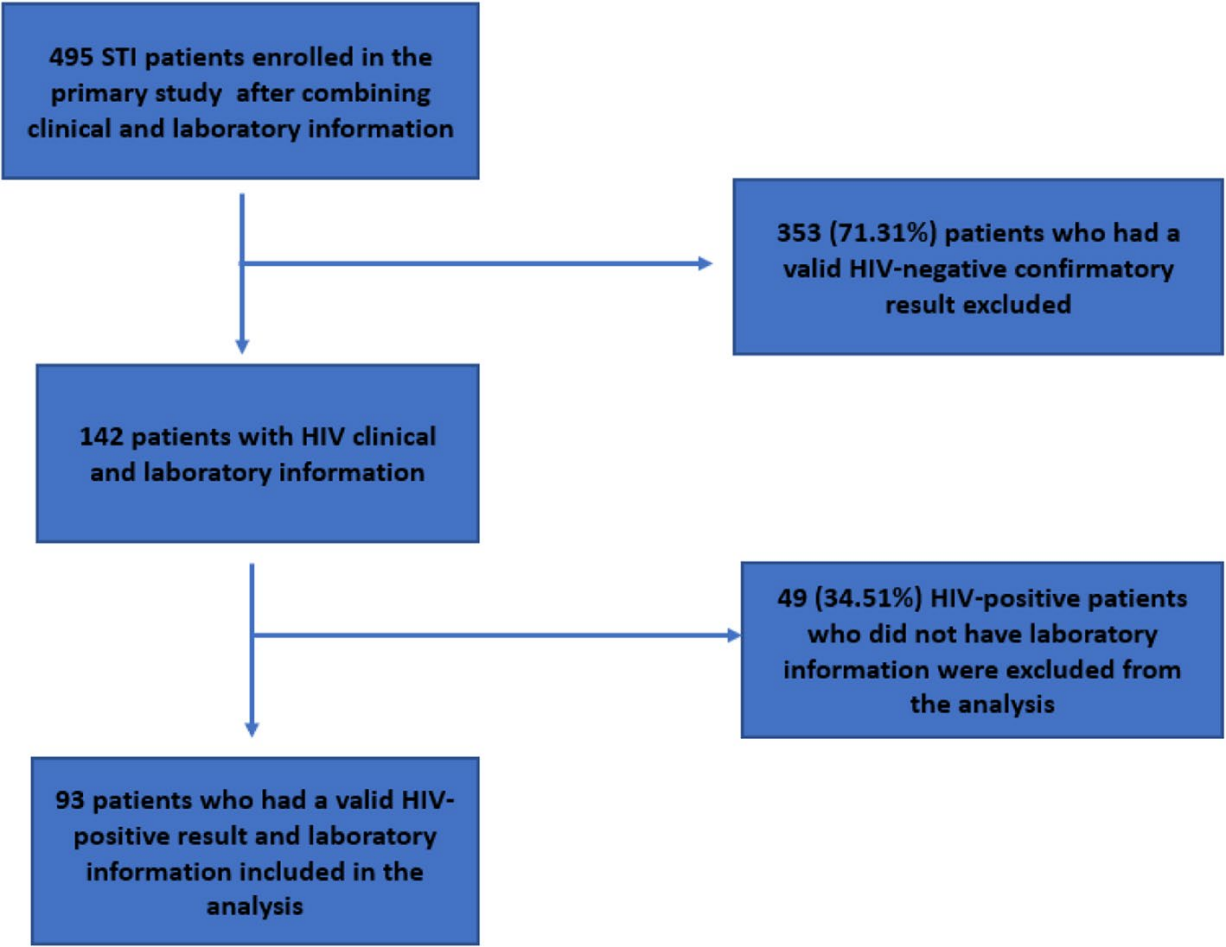
Flowchart showing stages of eligibility and exclusion of individuals included for the analysis

The primary study enrolled 495 individuals. Of these, 353 (71.3%) were HIV-negative and therefore excluded from the analysis, leaving 142 (28.9%) individuals who had positive HIV results. Of the remaining HIV-positive individual s, 49 (34.5%) were excluded because their records did not include VL data (the outcome of interest). This left 93 individuals i eligible for inclusion in the analysis (Fig. 1).

We did an analysis of the distribution amongst the HIV-positive individuals with (93) and without (49) VL data to determine selection bias as a result of missing VL data. Individuals without VL data were significantly younger than those with VL, but there were no additional significant differences between the two groups.

### Characteristics of HIV- positive individuals seeking STI services by ART status

Of the 93 individuals with VL data, the median age was 32 years (IQR 27–37). Overall, less than half of individuals were on ART (32, 34.4%) compared to 61 (65.6%) who were not on ART. Most individuals on ART were aged ≥25 years (90.6%) and male (65%). Overall, 75% of HIV-positive individuals seeking STI services did not use condoms at the last sexual encounter and 76% (71) had an unsuppressed VL (≥50 RNA copies/ml) (Table 1). Among those on ART, 3% reported practicing receptive anal sex compared to 11% among those not on ART. Among those with a casual sex partner, there was an equal number on ART (50%) compared to those not on ART.

**Table 1:** Demographic, behavioral and clinical characteristics of HIV-positive individuals attending STI services by ART use, Gauteng and Western Cape provinces, 2019

Characteristic	All individuals (*N* = 93)		On ART (*N* = 32)		Not on ART (*N* = 61)		*p*-value
	**n**	**%**	**n**	**%**	**n**	**%**	
**Age (years) (median, IQR)**	32	(27–37)	33	(27–38)	32	(27–37)	0.464
**Age (years)**							
< 25	11	(11.83)	3	(9.38)	8	(13.11)	
≥ ≥ 25	82	(88.17)	29	(90.63)	53	(86.89)	0.596
**Age at first sex:**							
< 15 years	10	(10.75)	3	(9.38)	7	(11.48)	
≥ ≥ 15 years	83	(89.25)	29	(90.63)	54	(88.52)	0.756
**Gender**							
Male	61	(65.59)	20	(62.50)	41	(67.21)	
Female	32	(34.41)	12	(37.50)	20	(32.79)	0.649
**Oral sex in the past 3 months**							
No	85	(91.40)	29	(90.63)	56	(91.80)	
Yes	8	(8.60)	3	(9.38)	5	(8.20)	0.847
Receptive anal sex in the past 3 months**							
No	85	(91.40)	31	(96.88)	54	(88.52)	
Yes	8	(8.60)	1	(3.13)	7	(11.48)	0.256
**Sex partner type:**							
Casual	18	(19.35)	9	(28.12)	9	(14.75)	
Regular	75	(80.65)	23	(71.88)	52	(85.25)	0.167
**Number of sex partners (last 3 months)**							
One partner	59	(63.44)	21	(65.63)	38	(62.30)	
Two or more partners	34	(36.56)	11	(34.38)	23	(37.70)	0.751
**Condom use at the last sexual encounter**							
Yes	23	(24.73)	11	(34.38)	12	(19.67)	
No	70	(75.25)	21	(65.63)	49	(80.33)	0.118
**STI syndrome, n (%)**							
Genital ulcer	26	(27.96)	12	(37.50)	14	(22.95)	
Male urethral	42	(45.16)	11	(34.38)	31	(50.82)	
Vaginal discharge	25	(26.88)	9	(28.13)	16	(26.23)	0.238
**Treatment without success for the same STI in the past 3 months**							
No	86	(92.47)	28	(87.50)	58	(95.08)	
Yes	7	(7.53)	4	(12.50)	3	(4.92)	0.188
**Previous STI in the last 12 months**							
No	77	(82.80)	27	(84.38)	50	(81.97)	
Yes	16	(17.20)	5	(81.97)	11	(18.03)	0.770
**Month since last HIV test**							
< 3 months	35	(37.63)	3	(9.38)	32	(52.46)	
≥ 3 months	58	(62.37)	29	(90.63)	29	(47.54)	< 0.001**
**Unsuppressed VL (≥ 1000 copies/ml)**							
Unsuppressed	61	(65.59)	12	(37.50)	49	(80.33)	
Suppressed	32	(34.41)	23	(62.50)	12	(19.67)	< 0.001**
**Unsuppressed VL ≥ 50 copies/ml**							
Unsuppressed	71	(76.34)	18	(56.25)	53	(86.89)	
Suppressed	22	(23.66)	14	(43.75)	8	(13.11)	< 0.001**

^**^Wilcoxon rank-sum test IQR-Interquartile range

The prevalence of having an unsuppressed VL was higher in females (80.3%) than males (68.8%), individuals aged <25 years (90.9%) compared to those ≥25 years (74.4%), those who first had sex at age <15 years (90.0%) compared to those who first had sex aged ≥15 years (74.70%), or those who did not use a condom during their last sexual encounter (81.4%) compared to those who used a condom (73.9%) and among individuals who were not on ART (86.9%) compared to those on ART (56.3%). The prevalence of having unsuppressed VL was higher among individuals with GUS (19/26) compared to those with either VDS or MUS (52/67).

### Factors associated with an unsuppressed VL

ART use was associated with a 33% lower prevalence of having an unsuppressed VL (adjusted prevalence rate ratio (aPRR= 0.67, 95% confidence interval [95% CI] =0.49–0.92). In the univariate regression, those aged <25 years (PRR =1.22, 95% CI= 0.97–1.53); and those who had first sex aged <15 years (PRR=1.20, CI=0.94–1.53) were associated with having unsuppressed VL. However, the females had higher likelihood of unsuppressed VL (PRR =1.17, CI=0.89-1.52) compared to males although the associations did not reach statistical significance. In a model adjusting for age, age at first sex and oral sex, none of the factors were significant (Table 2).

**Table 2:** Prevalence and factors associated with an unsuppressed viral load (VL ≥  ≥ 50 copies/ml) among HIV-positive individuals attending STI services, Gauteng and Western Cape, 2019, *N* = 93

Characteristic	VL ≥ 50 (n/N)	% with VL ≥ 50	Univariable PRR (95% CI)	*p*-value	Multivariable PRR (95% CI)	*p*-value
**Age category**						
≥ 25 years	61/82	74.39	Ref			
< 25 years	10/11	90.90	1.22 (0.97–1.53)	0.08*	0.27 (0.42–1.77)	0.17
**Age at first sex**						
≥ 15 years	62/83	74.70	Ref			
< 15 years	9/10	90.00	1.20 (0.94–1.53)	0.13*	0.50 (0.73–3.43)	0.48
**Gender**						
Male	49/61	68.75	Ref			
Female	22/32	80.33	1.17 (0.89–1.52)	0.25*	1.79 (0.88–3.63)	0.11
**Number of sex partners (last 3 months)**						
One partner	45/59	76.27	Ref			
Two or more	26/34	76.47	1.00 (0.79–1.26)	0.98		
**Oral sex**						
No	64/85	75.29	Ref			
Yes	7/8	87.50	1.16 (0.87–1.55)	0.56*	0.48 (0.84–2.75)	0.41
**Anal sex**						
No	65/85	76.47	Ref			
Yes	6/8	75.00	0.98 (0.64–1.49)	0.94		
**Sex partner type**						
Regular	58/75	77.33	Ref			
Casual	13/18	72.22	1.23 (0.52–2.89)	0.64		
**Condom use**						
No	54/70	81.43	Ref			
Yes	17/23	73.91	0.96 (0.73–1.26)	0.75		
**STI Syndrome**						
VDS/MUS	52/67	77.61	Ref			
GUS	19/26	73.07	1.09 (0.56–1.78)	0.74		
**Treatment of STI no success (3 months)**						
No	66/86	76.74	Ref			
Yes	5/7	71.43	0.93 (0.57–1.51)	0.77		
**Previous STI in the last 12 months**						
No	59/77	76.62	Ref			
Yes	12/16	75.00	72–1.33)	0.89		
**on ART**						
No	53/61	86.89	Ref			
Yes	18/32	56.25	0.65 (0.47–0.89)	0.008*		

^*^Variables with a *p*-value < 0.25 were selected for multivariable analysis

### Sub-group analysis for those on ART

Among those on ART, 21/32 (67.7%) reported excellent adherence defined as not missing any day of taking their treatment. Most individuals on ART (68.8%) reported sometimes taking their treatment correctly between 4 and 10 days a month.

All individuals <25 years (100%, 3/3) on ART had an unsuppressed VL compared to 51.7% (15/29) of individuals aged ≥25 years. Among individuals using ART, females were more likely to have an unsuppressed VL (58.3%, 7/12) compared to males (55.0%, 11/20). Individuals who reported having regular sexual partners (56.5%) had an unsuppressed VL compared to those who reported having casual sexual partners. Individuals with GUS had a higher prevalence of having an unsuppressed VL (58.33%) compared to individuals who had either MUS or VDS. There was a high likelihood of unsuppressed VL among individuals aged <25 years, those who had first sex at age <15 years and those who practiced individuals oral sex although the associations did not reach statistical significance. In a multivariable subgroup analysis limited to individuals on ART, individuals aged <25 years had a higher likelihood of having an unsuppressed VL (aPRR=1.94; 95% CI= 1.27–2.97) compared to those aged ≥25 years in a model adjusting for sex partner number and oral sex.

## Discussion

This study looked at factors associated with having an unsuppressed VL among HIV-positive individuals attending STI services in SA. The prevalence of having an unsuppressed VL was high regardless of ART status (76.0%). We found a low proportion of HIV-positive individuals seeking STI services on ART (34.4%), and low condom use (25.0%). A subgroup analysis among only those on ART showed that younger age and having multiple sex partners were associated with having an unsuppressed VL.

As expected, ART was protective against having an unsuppressed VL since ART use results in direct suppression of HIV VL. Correspondingly, it is consistent with studies that found that being on ART increases VL suppression [8, 9]. Additionally, the results highlighted that younger individuals aged <25 years had a higher risk of having an unsuppressed VL. Previously, studies that looked at age as a factor for unsuppressed VL similarly concluded that younger ages had a higher likelihood of VL non-suppression [8–10]. The prevalence of having an unsuppressed VL was higher in females (80.3%) than in males (68.8%). This contrasts with many studies where men had a higher likelihood of having an unsuppressed VL than women due to differences in health-seeking behaviour and ART adherence [9, 11–13]. In this study, the higher number of females or younger individuals with elevated VL may relate to different consent patterns for younger people and females (if for example males and older individuals could be more hesitant to participate if they do not know their HIV status / are not taking ART).

There was a low proportion of individuals on ART (34.4%) and high levels of having an unsuppressed VL defined as a VL ≥  ≥ 50 copies/ml (76.0%). Also, considering the ≥ 1000 copies/ml threshold, the rate of having an unsuppressed VL was 65.6% which did not reach the 3^rd^ 95 of 95–95-95 HIV prevention cascade benchmark and prevention goals. The low rates of ART and VL suppression are concerning since such individuals may pose a risk of transmitting STI or HIV infections to partners. High levels of having an unsuppressed VL may indicate gaps in the monitoring of ART adherence and/or clinical management of HIV-positive individuals seeking STI services. ART coupled with VL suppression have been the key intervention to reduce HIV transmission and improve the health of HIV-infected persons.

There low levels of reported condom use, is concerning which can increase the risk of HIV or STI transmission to uninfected partners among HIV-positive people. Similar findings showed from studies such as the PARTNER study from 14 European countries also found a high prevalence of condom-less sexual practices among HIV-positive individuals on ART seeking STI treatment [12]. HIV guidelines recommend consistent and correct use of condoms to reduce HIV transmission coupled with ART treatment [13]. ART can suppress VL to an undetectable level making HIV untransmittable (Undetected=Untransmittable). Low use of condoms can be prominent if partners are virally suppressed in this era of U=U. However, our findings suggest that this was not the case in our study population given the high prevalence of having an unsuppressed VL (76%). Consequently, more education and promotion of condom use among HIV-positive individuals, regardless of whether they are on ART, is needed to reduce STI rates and HIV acquisition. STIs are a marker of risky sexual behaviours which can range from having many sexual partners to no or inconsistent condom use and sexual debut at a young age which increases the transmission of HIV.

In a subgroup analysis among only those on ART, the prevalence of having an unsuppressed VL was high among females aged <25 years which may be due to non-adherence or no direct monitoring of treatment. Poor adherence amongst young adults on ART has been found in other studies with a higher likelihood of having an unsuppressed VL [14]. Having multiple sex partners was associated with low VL suppression among individuals seeking STI services who were on ART. Literature has equally highlighted an association between the failure to achieve VL suppression among individuals on treatment and increased risky sexual behaviours, including having multiple sexual partners [15, 16]. Despite low levels of ART coverage, being on ART showed a marked decrease in the prevalence of having unsuppressed VL.

### Limitations

The study contributes to existing data on factors associated with having an unsuppressed VL among HIV-positive individuals by ART use. However, our findings must be considered with some limitations. Firstly, the main outcome of having unsuppressed VL is laboratory-based which provided VL data for only HIV-positive individuals who had samples collected for laboratory examination. We might have missed HIV-positive individuals who did not consent to sample collection.. Secondly, we analysed self-reported data on the use of ART, previous STI symptoms, act type, sex partner number, age at first sex as well as being on ART among many others. Self-reporting may be biased due to recall and underreporting since sexual behaviour is confidential and thereby not easy to disclose. Thirdly, secondary data used had HIV-positive individuals attending STI services enrolled from two provinces which limit the generalizability of our findings to the rest of South Africa. We used secondary data where the primary study collected data appropriate for sentinel surveillance on STI individuals in An example is a question on condom use which was limited to the last sexual encounter and therefore did not capture whether earlier sexual encounters were condom-protected. The data did not have information on the duration of the current STI infection, only data on male circumcision was collected. Lastly, the study ended up with a small sample size, since we had to limit our analysis to individuals with a recorded VL as the main outcome which limited statistical power.

## Conclusion

VL suppression among HIV-positive individuals seeking STI services was low (24.0%) and fell short of the UNAIDS target of 95%. For those on ART, VL suppression was higher (43.80%) but still did not reach the 3^rd^ 95% target. The study has furthermore demonstrated that despite the national achievement of expanded HIV testing there is still a need to improve ART initiation and adherence to achieve VL suppression. There is a need for effective implementation of consolidated screening, prevention, and treatment approaches for HIV and STIs in clinical settings as recommended by the national guidelines. To achieve effective results, all individuals seeking STI service s must be tested for HIV (with consent), initiated for treatment if positive, and counseled about the importance of ART continuation and adherence. There is a need to keep individuals on treatment despite improved regimens and services providing ART. Wider scale up of self-testing and improving treatment response among the population at risk. 

 Therefore, intensified targeted HIV/ STI programs and ART support can help help reach HIV treatment coverage and targets. Improved implementation of integrated HIV-STI prevention, testing, and treatment will efficiently and effectively reach high-risk populations and help reduce transmission

## Data Availability

Data from this study are available from the Centre for HIV/ STI at the National Institute for Communicable diseases (NICD), South Africa, but restrictions apply to the availability of these data. However, data is available from Marceline Mapiye upon reasonable request and with permission of the Centre for HIV/ STI.

## Abbreviations

ART: Antiretroviral therapy
CI: Confidence Interval
GUS: Genital ulcer syndrome
Human HIV: Immunodeficiency Virus
IQR: Interquartile range
MUS: Male urethritis syndrome
NDoH: National Department of Health
OR: Odds ratio
PRR: Prevalence rate ratio
STI: Sexual Transmitted Infection
SDG: Sustainable development goals
UNAIDS: United Nations AIDS
U: U
Undetected: Un-transmissible
UTT: Universal test and treat
VDS: Vaginal discharge syndrome
VL: Viral Load
WHO: World Health Organization
<: Less than
>: Greater than
